# Vascular Endothelial Growth Factor (VEGF) as a Biomarker for Cancer-Associated Venous Thrombosis: A Meta-analysis

**DOI:** 10.1055/a-2513-4381

**Published:** 2025-02-10

**Authors:** Alison M. Brown, Sophie Nock, Kathryn Musgrave, Amanda J. Unsworth

**Affiliations:** 1 Department of Blood Sciences, The Newcastle upon Tyne Hospitals NHS Foundation Trust, United Kingdom; 2 Department of Life Sciences, Faculty of Science and Engineering, Manchester Metropolitan University, Manchester, United Kingdom; 3 Haematology Department, The Newcastle upon Tyne Hospitals NHS Foundation Trust, United Kingdom; 4 Thrombosis Collective, Leeds Institute of Cardiovascular and Metabolic Medicine, Faculty of Medicine and Health, University of Leeds, Leeds, United Kingdom

**Keywords:** VEGF, cancer, thrombosis, biomarker

## Abstract

Cancer-associated thrombosis affects between 1 and 20% of all patients diagnosed with cancer and is associated with significant morbidity and a poorer prognosis. Risk assessment scores exist which include the measurement of biomarkers, and which aim to identify patients at a higher risk of developing thrombotic events, but these are poor predictors and rarely used in routine clinical practice.

VEGF is a potent angiogenic factor, produced by tumour cells, and released by platelets and is essential for tumour growth and progression. It also plays a role in the promotion of thrombosis through platelet activation and adhesion, and by inducing the expression of tissue factor. Therefore, the potential of VEGF to be used as a biomarker to predict cancer-associated thrombosis requires further investigation.

This study reviewed the published literature to determine whether circulating VEGF levels are associated with increased risk of venous thromboembolism in patients with cancer.

PubMed and OVID databases were systematically searched according to PRISMA guidelines for relevant papers using the keywords “cancer” AND “thrombosis” AND “VEGF” up to July 2023. Inclusion and exclusion criteria were applied.

Seven papers (1,528 participants) were identified and included in the meta-analysis, three of which (922 participants) measured VEGF before a thrombotic event, and the remaining four (606 participants) measured VEGF at the time of the thrombosis. Our results showed that although plasma and serum VEGF tended to be higher in those who subsequently developed thrombosis than those who did not (mean difference 70.2 pg/mL for serum, and 11.44 pg/mL for plasma VEGF, 95% CI −2.39–25.73, *p* = 0.10), this was not found to be statistically significant. However, analysis of VEGF following blood sampling at the time of thrombosis showed a stronger statistically significant association between increased VEGF levels and presence of thrombosis (mean difference 117.02 pg/mL for serum, and 116.6 pg/mL for plasma VEGF, 95% CI 55.42–190.82, *p* = 0.0004).

Based on current studies, whilst it is increased at the time of thrombosis, VEGF is not effective as a predictive biomarker of CAT.

## Introduction

Cancer-associated thrombosis (CAT) affects up to 20% of patients with cancer and is associated with a poorer prognosis. 1 2 3 The use of low-dose anticoagulation (thromboprophylaxis) has been shown to not only reduce the risk of venous thrombosis but also increases the risk of bleeding, 4 which complicates the clinical picture and does not allow routine thromboprophylaxis to be given to all people with cancer in the outpatient setting. 5

Clinicians need to target the use of thromboprophylaxis and offer it to those at highest risk of thrombosis. A way of predicting those who are a higher risk of developing a venous thromboembolism (VTE) has been a long sought-after clinical decision-making tool.

To address this, numerous risk assessment scores have been proposed, some of which use circulating levels of biomarkers at the time of diagnosis of the cancer. The most validated is the Khorana score 6 which uses the major parameters of a full blood count—haemoglobin, white cell count and platelets, along with patient factors such as cancer site and body mass index (BMI)—to determine the likelihood of a thrombosis. The Vienna CATS score 7 goes further and has added two additional biomarkers, namely, soluble P-selectin and D-dimers, to predict those individuals at a greater risk of thrombosis.

However, whilst these prediction scores demonstrate a strong association with VTE, in that those assigned to a high-risk category are more likely to develop a thrombosis, these scores can identify only a proportion of all individuals who will develop a thrombosis 3 and have limited discriminatory power. 8 About 90% of patients who are in either the intermediate- or high-risk categories based on the Khorana score do not develop a thrombosis after 6 months. 8 Therefore, these risk assessment scores need to be improved to truly distinguish the patients who are at a higher risk of developing a thrombosis, and who would benefit from receiving thromboprophylaxis.

Vascular endothelial growth factor (VEGF or VEGF-A) is a potent angiogenic factor 9 that is also thought to promote thrombosis. Angiogenesis, the formation of new blood vessels, is essential for the growth, invasion, progression, and metastasis of tumour tissue. 10 As a result, VEGF has been shown to be overexpressed in breast, colorectal, lung, pancreatic, ovarian, and cervical cancers. 1 10

In health and disease, VEGF is expressed on the surface of many different cell types, including monocytes, endothelial cells, lymphocytes, and granulocytes, 1 11 but it is thought that VEGF levels in these cells are higher in cancer than in healthy individuals. 12 Platelets, cells that are essential for thrombosis, are also rich in VEGF, which is stored within their alpha granules. 1 In cancer, both radiotherapy and chemotherapy have been shown to increase VEGF within tumours. 13

Despite its association with both cancer and thrombosis, the predictive value of VEGF, in CAT events, is less well defined.

Herein we present a meta-analysis of previously published data to assess the predictive potential of VEGF in CAT.

## Methods

### Search Strategy and Eligibility Criteria

This meta-analysis complies with the standard of Preferred Reporting Items for Systematic Reviews and Meta-Analyses (PRISMA). 14

A literature search was performed using two databases, PubMed and OVID, until 9 July 2023. Papers were included only if published after the year 2000. This time frame was chosen to represent recent research. One paper (Musolino et al, 2002 ^21^ ) was found by examining the references of another paper.

Keywords included: ‘cancer’, ‘thrombosis’, and ‘VEGF’. The following search terms were also used: (‘cancer’ OR ‘neoplasms’) AND (‘VEGF’ OR ‘vascular endothelial growth factor’ OR ‘vascular endothelial growth factors’ [Mesh Major Topic] OR ‘vascular permeability factor’ OR ‘biomarkers/analysis’ [Mesh] OR ‘biomarkers/blood’ [Mesh]) AND ‘thrombosis’ OR ‘vte’ OR ‘Thrombosis/blood’ [Mesh] OR ‘Thrombosis/complications’ [Mesh] OR ‘Thrombosis/diagnosis’ [Mesh] OR ‘Thrombosis/epidemiology’ [Mesh] OR ‘Thrombosis/etiology’ [Mesh] OR ‘Thrombosis/immunology’ [Mesh] OR ‘Thrombosis/pathology’ [Mesh]).

Inclusion criteria: (1) patients with cancer being studied, (2) studies reporting either plasma or serum VEGF levels in patients with cancer, in both those with a thrombosis and those without, quantitatively, (3) VEGF measured before or during the thrombotic event, (4) adults over the age of 18 studied, (5) full text available, and (6) studies written in English.

Exclusion criteria: (1) Paediatric population being studied, (2) review article, case report, or conference abstract, (3) cell lines and not patients studied, (4) full text not available, (5) not written in English, (6) study did not have figures for thrombosis and no thrombosis, and (7) subjects studied were not humans.

This study focussed on venous thrombosis, including unusual site thrombosis such as portal vein thrombosis. The references of relevant studies and review articles were also studied and checked for relevance to identify additional studies. Two additional authors (SN and AU) validated the search and assessed the articles and abstracts.

### Data Extraction and Quality Assessment

Following the inclusion and exclusion criteria above, and data selection, studies were further examined for suitability. Data extraction was performed by AB. All VEGF values were converted to pg/mL irrespective of the values used originally in the study to allow an easier comparison between them. Two studies (Kirwan et al, 2008 15 ; Kirwan et al, 2009 16 ) quoted VEGF values as µg/mL, representing a 10 ^6^ difference between these results, and other comparable studies. Attempts were made to verify these values. As the values given were comparable to those which were given in pg/mL, and based on the sensitivity and range of the enzyme-linked immunosorbent assay (ELISA) used (9 pg/mL), these values were subsequently assumed to be pg/mL and are represented as such.

Studies where thrombosis had already occurred at the sampling point were also included. All studies measured VEGF by an ELISA method. Further details of the studies were included, and their design are shown in Table 1 .

**Table 1:** Summary of the study designs included in meta-analysis (* denotes not included in forest plots due to lack of availability of data)

Study (year published)	Geographical location of study	Study design	Total number of participants	Cancer type(s) and stage	Type of thrombosis	Control group?	Newcastle-Ottawa Quality Assessment Score	VEGF biomarker measured
** Dogan et al (2006) 10**	Turkey	Prospective cohort	31	All types and stages	Venous	51 matched pairs (all had cancer)	7	Serum VEGF
** Kim et al (2004) 22**	Korea	Prospective cohort	52	Hepatocellular carcinoma (HCC), all stages	Portal vein	30 healthy, 26 liver cirrhosis	9	Serum VEGF, and serum VEGF per platelet count
** Kirwan et al (2008) 15**	United Kingdom	Prospective cohort	123	Breast, early and advanced stages	Venous	68 healthy controls	9	Plasma VEGF
**Kirwan et al** ** (2009) 16**	United Kingdom	Prospective cohort	123	Breast, early and advanced stages	Venous	68 healthy controls	9	Plasma VEGF, serum VEGF and platelet release of VEGF
Li et al (2004)* 9	China	Prospective cohort	45	Hepatocellular carcinoma (HCC), all stages	Portal vein	17 healthy, 20 benign liver lesions	9	Plasma VEGF
** Malaponte et al (2015) 24**	Italy	Retrospective case-control	385	All types and stages	DVT only	100 healthy controls	7	Plasma VEGF
Musolino et al (2002)* 21	Italy	Retrospective cohort	55	Myeloproliferative neoplasms	All	20 healthy	4	Plasma VEGF
Nazari et al (2019)* 20	Austria	Prospective cohort	76	Glioma	Venous	No	7	Unclear if plasma or serum VEGF
** Posch et al (2016) 11**	Austria	Prospective cohort	804	All types and stages	Venous	No	7	Plasma VEGF
** Ramadan et al (2021) 23**	Egypt	Prospective cohort	87	Hepatocellular carcinoma (HCC), all stages	Portal vein	No	7	Serum VEGF

Abbreviations: DVT, deep vein thrombosis; VEGF, vascular endothelial growth factor.

Patient characteristics from the included studies are shown in Table 2 .

**Table 2:** Summary of the patient characteristics used in meta-analysis where available

Study (year published)	Total number of participants	Age of participants in years (range) (Mean or median)	Sex	Body mass index (BMI) (range)	Blood cell count: Platelets (×10 ^9^ /L) Haemoglobin (g/L) White blood cell count (×10 ^9^ /L)	D-dimer levels (ng/mL) (range)	Fibrinogen level (g/L)	Cancer type(s) and stage
Dogan et al (2006) 10	31	56.74 +/− 16.06 (mean)	Male = 13, Female = 18	Not stated	Not stated	960.71 +/− 1,066.85	Not stated	All types and stages
Kim et al (2004) 22	52	57 (35–80) (median)	Male = 39, Female = 13	Not stated	Platelet count: 130 (76.4–217.3)	Not stated	Not stated	Hepatocellular carcinoma (HCC), all stages
Kirwan et al (2008) 15	123	52 (31–78) (median)	Female = 123	Not stated	Platelet count: 314.3 (287.2–325)	1,618.6 (979–2,676.1) with thrombosis 815.3 (707.8–989.3) without thrombosis	3.6 (3.3–3.8) with thrombosis 4.9 (3.0–6.9) without thrombosis	Breast, early and advanced stages
Kirwan et al (2009) 16	123	52 (31–78) (median)	Female = 123	Not stated	Platelet count: 314.3 (287.2–325)	1,618.6 (979–2,676.1) with thrombosis 815.3 (707.8–989.3) without thrombosis	3.6 (3.3–3.8) with thrombosis 4.9 (3.0–6.9) without thrombosis	Breast, early and advanced stages
Li et al (2004)* 9	45	50 (29–77) (mean)	Male = 37, Female = 8	Not stated	Not stated	Not stated	Not stated	Hepatocellular carcinoma (HCC), all stages
Malaponte et al (2015) 24	385	62 +/− 9 (mean) no DVT 64 +/− 10 (mean) with DVT	Male = 185, Female = 200	25.85 +/− 8.3	Not stated	Not stated	413.7 +/− 87.7 with thrombosis 404.2 +/− 71.1 without thrombosis (Units not stated)	All types and stages
Musolino et al (2002)* 21	55	60 (median)	Male = 17, Female = 38	Not stated	Not stated	Not stated	Not stated	Myeloproliferative neoplasms
Nazari et al (2019)* 20	76	54 (46–67) (median)	Male = 41, Female = 35	Not stated	Not stated	Not stated	Not stated	Glioma
Posch et al (2016) 11	804	63.1 (54.2–69.2) (median)	Male = 371, Female = 433	25.0 (22.3–28.1)	Platelet count 245 (199–302) Haemoglobin 131 (120–141) White blood cell count 7.2 (5.7–9.4)	710 (360–1,320)	3.94 (3.25–4.83)	All types and stages
Ramadan et al (2021) 23	87	61.93 +/− 6.99 (mean) Group 1, 64.42 +/− 8.87 Group 2	Male = 68, Female = 19		Platelet count 141.7 +/− 80.2 Haemoglobin 112.1 +/− 24.9 White blood cell count 6.90 +/− 3.77	Not stated	Not stated	Hepatocellular carcinoma (HCC), all stages

Abbreviation: DVT, deep vein thrombosis.

Note: Chemotherapy regimens and antithrombotic treatments not included due to a lack of information.

In instances where research papers contained qualitative findings and no comparable quantitative data, the studies were included in a qualitative manner.

Two authors (AB and SN) evaluated the quality of the studies independently. If a disagreement occurred, a third investigator made the final decision. Quality assessment of the included studies was performed using the Newcastle-Ottawa score (NOS). 17 The Agency for Healthcare Research and Quality's (AHRQ) 11-item criteria were used to evaluate each of the studies. A score of 6 or more was considered to indicate good quality.

### Statistical Analysis

The association of VEGF with CAT was evaluated by calculating the mean and SD values for plasma and serum VEGF levels for each study. Therefore, in this meta-analysis, studies looking at plasma and serum levels of VEGF have been separated into different forest plots to allow easier comparisons to be drawn. Currently, there is no consensus on which is the better VEGF parameter to measure.

Meta analysis of the mean difference for random effects was performed using Rev Man software. Random effects as opposed to fixed effects were used due to high heterogeneity between included studies. Heterogeneity between the included studies was tested using the Rev Man software and I ^2^ values. The chosen statistical significance threshold was set at *p* <0.05.

The risk of bias for this meta-analysis was assessed using the ROB-ME tool (Risk Of Bias due to Missing Evidence in a meta-analysis). 18 This tool identified that there was a low risk of bias with this meta-analysis.

## Results

### PRISMA Protocol

**Fig. 1:**
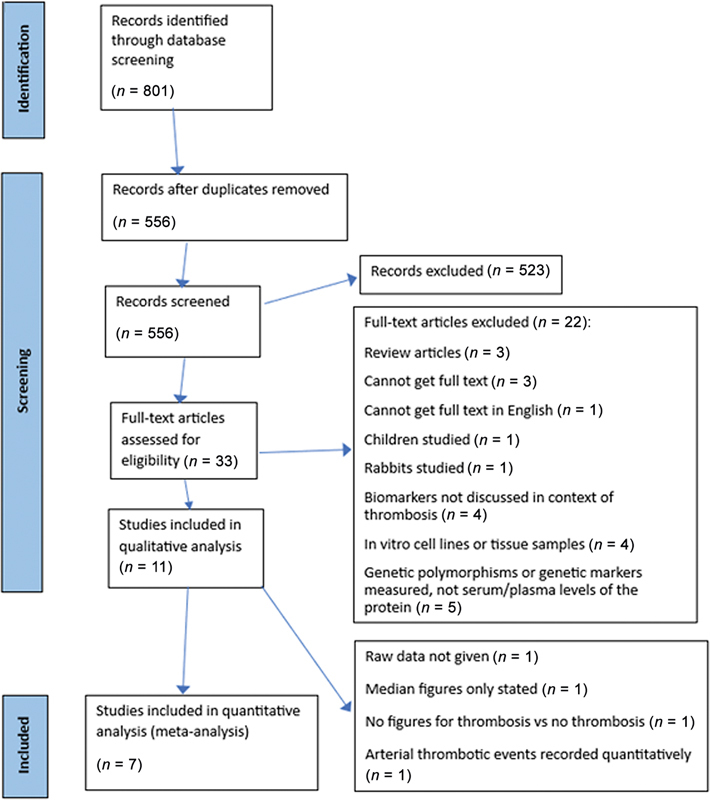
Flow diagram of the inclusion and exclusion procedures. PRISMA, Preferred Reporting Items for Systematic Reviews and Meta-Analyses.

A total of 801 records were identified through screening of two databases: PubMed and OVID. After duplicates were removed, 556 papers remained. Review of the paper title and abstract reduced the number of papers to 33. For these remaining papers the full text was accessed and assessed for eligibility. Once the inclusion and exclusion criteria were applied, 11 records remained. A further study was excluded as it mainly described arterial thrombotic events (Cacciola et al, 2002 19 ). Of the remaining, only seven of those could be included in the meta-analysis due to the lack of data ( Fig. 1 ). The remaining three are still included in the meta-analysis but qualitatively rather than quantitatively. This is due to the raw data either not being available (Nazari et al, 2019 20 ) or presented in a different format which did not allow inclusion in the forest plots (only a median value was provided by Li et al, 2004, 9 and Musolino et al, 2002 21 did not present the figures for thrombosis and no thrombosis as two separate populations). Attempts were made to contact the authors where data were missing, though in two cases the papers were published 20 and 22 years ago.

The main characteristics of the seven papers used for the meta-analysis, plus the three used qualitatively, are summarized in Table 1 .

### Patient Characteristics

The overall population included in the meta-analysis consisted of 1,528 participants, 213 of which were patients with cancer who were affected by thrombosis. The remaining 1,315 were patients with cancer who were not affected by thrombosis, representing a 14% rate of CAT in the study population. This figure agrees with the widely reported rates of CAT. 1 2 3 In some cases, the nature of the thrombosis was recorded, but in others it was not.

All types of cancer and all stages of the disease were represented in the data studied. The seven studies represent a wide geographical area ( Table 1 ) and the median age of participants across the seven studies was 57.82 years. Individual studies' participant characteristics are shown in Table 2 .

### Quality Assessment and Risk of Bias

Quality assessment of the 11 included studies was performed using the NOS scale. 17 Of the 11 studies 10 were assessed to have scores greater 6 and therefore of good quality, with the remaining study (Musolino et al, 2002 21 ) considered to be of moderate quality (score of 4).

### Meta-analysis of VEGF Levels on Thrombotic Events in Cancer

#### VEGF Levels at the Time of Thrombosis are Increased in Cancer Patients

**Fig. 2:**
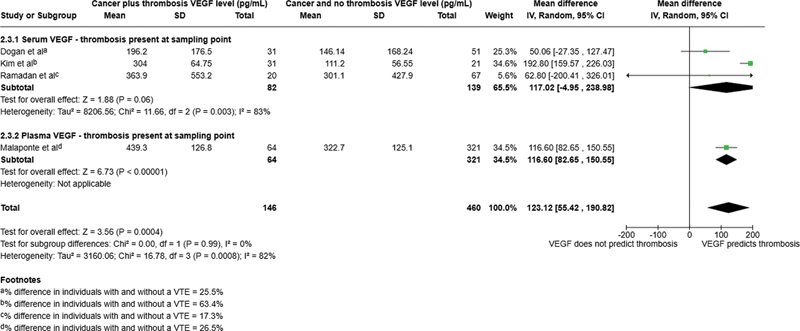
Forest plot for vascular endothelial growth factor (VEGF) levels among cancer-associated thrombosis and patients with cancer and no thrombosis.

Four studies, with 606 patients (146 with thrombosis), assessed VEGF levels at the time of the thrombotic event, three analyzed serum VEGF levels (Dogan et al, 2006, 10 Kim et al, 2004, 22 Ramadan et al, 2021 23 ), and one study analyzed plasma VEGF levels (Malaponte et al, 2015 24 ). Our analysis of the four studies identified significantly higher levels of VEGF in patients with thrombosis versus those patients without (mean difference 123.12 pg/mL, 95% CI 55.42–190.82, *p* = 0.0004) ( Fig. 2 ). Heterogeneity was assessed with a I ^2^ value of 82%. All four papers demonstrated that VEGF significantly rises at the time of a thrombotic event, with the percentage difference in VEGF levels between those with and without thrombosis of 17.3 and 63.4% across the four studies.

These findings are further supported by the work of Musolino et al 21 who showed that increased plasma VEGF levels were seen in patients with myeloproliferative neoplasms who had had a thrombotic event within the preceding month, and by the work of Li et al 9 who also showed that the presence of portal vein thrombosis in patients with hepatocellular carcinoma was associated with a higher plasma VEGF level.

Taken together these findings indicate a positive association of VEGF levels with thrombosis in cancer patients and identifies increased VEGF as a marker of CAT at the time of thrombosis.

#### VEGF Levels Prior to a Thrombotic Event are not Associated with Cancer-Induced Thrombosis

**Fig. 3:**
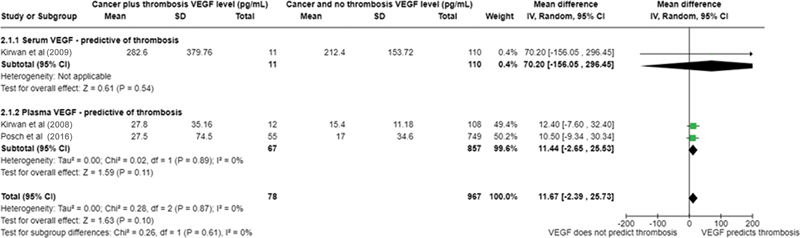
Forest plot for vascular endothelial growth factor (VEGF) levels, collected prior to thrombosis among cancer-associated thrombosis and patients with cancer and no thrombosis.

Having identified an association of VEGF levels with thrombosis post thrombotic event, we analyzed the three remaining studies, which measured VEGF levels prior to thrombotic event occurring, to determine whether VEGF could be used as a predictive biomarker of thrombosis. Three studies involving 922 participants examined the role of VEGF as a predictor of thrombosis (serum VEGF, Kirwan et al, 2009 16 plasma VEGF, Kirwan et al, 2008 15 and 2009 16 —data only included once—and Posch et al, 2016 11 ). The 3-month cumulative incidence of VTE in the Kirwan et al studies' population was 9.8%, whilst the 6-month cumulative incidence in the Posch et al study's population was 5.0%. Analysis of data from these studies show that whilst pre-event plasma VEGF or serum VEGF levels are higher in patients who go on to experience CAT there is no significant difference in VEGF levels between patients who develop thrombosis versus those who do not (mean difference 11.68 pg/mL, 95% CI −2.39–25.73, *p* = 0.10; Fig. 3 ). Heterogeneity was assessed, giving an I ^2^ value of 0%; this is possibly due to the papers included.

These findings are further supported by the work of Nazari et al, 20 which also showed no association of serum VEGF levels and the prediction of VTE in patients with glioma (hazard ratio per double increase: 0.995, 95% CI 0.640–1.548, *p* = 0.983).

Taken together these observations indicate that whilst VEGF levels are increased in cancer patients at the time of thrombosis ( Fig. 2 ) VEGF levels in cancer patients are not predictive of thrombosis.

## Discussion

Cancer is the uncontrolled proliferation of genetically aberrant cells, which is a leading cause of death throughout the world. It can occur in any tissue of the body, including the blood. For proliferation of the cancer cells to take place, certain conditions need to be in place, one of which is the ability for angiogenesis to occur, which is the formation of new blood vessels. 1 VEGF is a potent angiogenesis stimulator, and therefore we would expect VEGF to be raised in patients with cancer. 1

Compared to the general population, patients with cancer are at an increased risk of developing a thrombosis; between 1 and 20% of patients develop this complication, which is associated with a higher mortality rate. 1 2 3 Whilst both venous and arterial thrombotic events can occur in CAT, the incidence of VTE is widely considered to be equivalent to the incidence of CAT in patients diagnosed with cancer.

VEGF is raised in patients with cancer 1 10 12 and is thought to play a role in thrombosis 1 by promoting both the release of tissue factor, and platelet activation and adhesion. 11

Tissue factor, released from endothelial cells, is one of the main initiators of coagulation. 1 11 It may also play a role in angiogenesis, by upregulating VEGF, and downregulating the angiogenesis inhibitor thrombospondin, 25 26 a mechanism which is independent of coagulation activation. 25 27

Platelet adhesion and activation are involved in the thrombotic process. Activated platelets release further VEGF from their alpha granules 11 into the circulation enhancing thrombosis via these mechanisms. Platelets can also act as a transporter of tumour-originated VEGF, 28 further contributing to tumour angiogenesis and progression, as well as the risk of thrombosis.

Therefore, we hypothesized that VEGF shows excellent theoretical potential to be used as a biomarker for CAT. In this analysis we investigated whether plasma or serum VEGF levels are associated with thrombotic events in cancer patients, pre and post thrombosis.

Seven papers (six patient cohorts) were included in this meta-analysis. The findings presented here indicates that VEGF levels are increased at the time of a thrombotic event, indicating VEGF may play a role during a thrombotic event, in addition to its role in the pathogenesis of a malignancy, but it does not appear to be predictive of CAT/thrombosis.

Our meta-analysis included four studies where the thrombosis was present at the blood sampling point, to determine whether VEGF was associated with thrombus formation. All of these studies showed increased mean differences between patient groups who had a thrombosis versus those who did not ( *p* = 0.0004). These findings were further supported by the work of Musolino et al 21 and Li et al, 9 which demonstrated increased plasma VEGF in patients with thrombosis versus those with no thrombosis, but whose data were not compatible to be included in our forest plots analysis. Taken together these findings demonstrate that VEGF levels are significantly increased and associated with the presence of thrombosis in patients with cancer.

Activated platelets release VEGF, 11 and therefore it is not unexpected that VEGF levels were observed to be increased at the time of a thrombosis. Platelet activation is an essential part of primary haemostasis, which is required in the formation of a thrombus. VEGF is also found in higher levels in patients with cancer compared to healthy controls, 1 due to ongoing angiogenesis required for tumour growth and survival. 1 Interestingly Musolino et al 21 showed that in patients with myeloproliferative neoplasms increased plasma VEGF levels were seen up to 1 month post thrombotic event, possibly indicating a state of platelet hyperactivation and/or indicating a more global contribution of VEGF to thrombosis.

Having identified an association of VEGF with CAT at the time or post thrombosis, this meta-analysis set out to investigate whether VEGF can be used as a biomarker to predict thrombosis. Three studies identified by our search strategy collected blood samples for VEGF level measurement from cancer patients before thrombosis had occurred. The 3-month cumulative incidence of VTE was 9.8% for the Kirwan studies, 15 16 and the 6-month cumulative incidence in the Posch et al study population was 5.0%. This reflects typical CAT incidence, 1 2 3 and the two study populations' characteristics, as the Kirwan et al's studies include exclusively breast cancer patients associated with a higher risk of VTE, whereas Posch et al studied a variety of cancer types, with various differing risk profiles. Although all three studies showed a trend towards higher levels of VEGF in those patients who subsequently developed a thrombosis versus those who did not, this difference was not statistically significant ( *P* -value of 0.10). There are many reasons for this, including not knowing how long prior to the thrombotic event the samples were taken for example, which we hypothesize may impact the study's conclusions. Posch et al, 11 for example, followed patients for thrombotic events for 2 years following initial sampling as part of the large Vienna CATS Study, so it not inconceivable that VEGF would not be raised up to 2 years before a thrombotic event occurred. The work of Nazari et al 20 was also part of the same study and so the same conclusions can be drawn. In contrast, the two remaining studies, Kirwan et al, 2008 and 2009, 15 16 which used plasma and serum samples collected from the same cohort of 123 patients (120 for plasma, and 121 for serum), only followed patients for 3 months after blood sampling. These differences in follow-up time may be confounding the results. In addition, different cancer types were studied, at different stages, which may also be impacting the findings. It is also difficult to compare studies as plasma 15 and serum 16 VEGF levels were included from two publications that include the same patient population, which inevitably leads to bias. Overall, the lack of independent studies will have had an impact on the results obtained and highlights that further work in this area is required.

As part of this meta-analysis, we included studies measuring VEGF from both serum and plasma. This has consequences for our interpretation as serum and plasma VEGF have very different normal reference ranges. In this respect study by Malaponte et al 24 appears to be an outlier with the measurement of plasma VEGF recording VEGF levels much higher than the other groups also measuring these biomarkers, even in those individuals with no thrombosis. The reasons for this are unclear. However, the percentage difference in mean plasma VEGF values between individuals with and without a VTE was 26.5% in this study, which is comparable to that of other studies in the same category (25.5% in Dogan et al, 10 17.3% in Ramadan et al, 23 with Kim et al 22 being an outlier with a 63.4% difference). Therefore, all studies show that VEGF levels are higher in those with a thrombosis compared to those without.

Normal plasma and serum VEGF reference ranges differ significantly, with the serum level being 10 to 15 times higher than that of the plasma level (D'Souza et al 29 ). This is because the platelets will have become activated during centrifugation in the serum sample, but they remain intact in plasma samples due to the presence of anticoagulant in the sample tube. Serum VEGF analysis therefore gives a measure of how much VEGF there is in platelets, whereas plasma VEGF analysis does not, and instead represents VEGF released from platelets which is indicative of platelet activation.

By examining the forest plots, we can see that the measurement of serum VEGF is much more variable than that of plasma, and this is possibly affecting the significance of our findings. The difference in the values could also explain why serum VEGF was found to be associated with occurrence of thrombosis but not found to be predictive of thrombosis. Activated platelets secrete VEGF, indicating that they are prothrombotic, and therefore a thrombosis may occur. However, by analyzing a serum sample, where these ‘naturally- activated’ platelets are present, plus those platelets ‘artificially-activated’ by centrifugation, it is unlikely that we are truly representing the predictive value of VEGF measurement in serum samples. Plasma samples may therefore give a more accurate representation of the predictive value of VEGF in thrombosis in patients with cancer, and further studies are therefore needed to investigate this.

VEGF is a potent angiogenic factor that has been shown to be overexpressed in breast, colorectal, lung, pancreatic, ovarian, and cervical cancers, 1 10 where it promotes the formation of new blood vessels, and is essential for the growth, invasion, progression, and metastasis of tumour tissue. 10 Several of the studies included in this analysis demonstrated increased VEGF levels in cancer patients versus healthy controls. 9 15 16 21 22

VEGF levels also increase as a cancer develops. Patients with more advanced stages of cancer therefore can have higher levels of VEGF. 30 In the studies examined, this was acknowledged by all, but not considered with regards to the VEGF level and reported thrombosis rates. However, Dogan et al 10 matched controls according to cancer stage, which showed that those who experienced VTE still had higher VEGF levels than the matched controls, suggesting that the thrombotic process was an additional factor for an increase in VEGF levels. Posch et al 11 also addressed this, using multivariable analysis to adjust for tumour stage in their analysis, and showed that the association between VEGF and risk of VTE prevailed after adjustment.

The role of VEGF in initiating thrombus formation is also not well established. There is little to no evidence to suggest that VEGF alone can trigger thrombotic events, which may explain why our analysis found it not to be predictive of thrombosis. It is possible, however, that VEGF plays a role along with other prothrombotic factors to initiate thrombus formation. 6

Given the association of increased VEGF levels at the time of, or after, the thrombotic event, some consideration should be made as to whether adding VEGF as a biomarker to an existing risk-assessment model (RAM) could be useful. Other biomarkers such as D-dimer levels are already part of the Vienna CATS score, 7 with strong evidence available demonstrating increased D-dimer levels associated with both current and future thrombotic events. 31 32 33 34 Interestingly, the Kirwan studies (2008) show significantly higher D-dimer levels in patients who subsequently went on to experience a VTE versus those who did not (1,655 (834–3,273) ng ml ^−1^ vs. 727 (631–836) ng ml ^−1^ , *P* = 0.003); in the same cohort, VEGF tended to be higher, but this difference was not statistically significant.

At this time, our analysis of predictive studies demonstrates that there is not sufficient evidence that VEGF can be used to predict CAT independently. However, it is possible that VEGF levels may increase predictive capacity in combination with other established markers and risk scores, such as cancer type, 6 7 35 BMI, 6 7 35 and D-dimers, 7 or alongside other novel biomarkers such as soluble P-selectin. 7 36 The study by Posch et al 11 demonstrated a positive interaction between soluble VEGF levels and D-dimer, indicating that the predictive potential of VEGF might be enhanced in combination with D-dimer, particularly in individuals with high levels of both biomarkers. Further investigation and studies are required.

## Conclusion

We present here a meta-analysis approach to investigate whether VEGF has the potential to be used as biomarker for CAT. We identify that high plasma and serum VEGF levels are associated with current thrombosis in samples taken at the time of or post thrombotic event; however, plasma and serum VEGF levels were not found to be associated with or predictive of thrombosis when collected prior to thrombotic events in cancer patients. In the future, more prospective cohort studies in specific cancer types and stages are needed to ascertain whether VEGF could be used as a predictive biomarker of CAT.

## Notes

**What is Known on This Topic?**

- Patients with cancer are at an increased risk of developing a thrombosis, which are associated with a poorer prognosis.
- Prediction scores for cancer-associated thrombosis exist but although they have a strong association with VTE in cancer they typically have moderate to poor discrimination.

**What Does This Paper Add?**

- Vascular endothelial growth factor (VEGF) is increased at the time of thrombosis.
- Although increased at the time of thrombosis, further work is required to determine if a rise in VEGF levels could predict a thrombotic episode.
